# Surveillance of single nucleotide polymorphisms correlated to macrocyclic lactone resistance in *Dirofilaria immitis* from client-owned dogs across the United States

**DOI:** 10.1016/j.ijpddr.2025.100604

**Published:** 2025-08-05

**Authors:** Emily Curry, David Tack, Jessica Rodriguez, Danielle Brehm-Lowe, John Letherer, Megan Lineberry, Roger Prichard, Tobias Clark

**Affiliations:** a Zoetis, Veterinary Medicine Research and Development, 300 Portage Street, Kalamazoo, MI, 49007, USA; b Eurofins Lancaster Laboratories, 300 Portage Street, Kalamazoo, MI, 49007, USA; c Institute of Parasitology, McGill University, 21111 Lakeshore Road, Sainte Anne de Bellevue, QC, H9X 3V9, Canada

**Keywords:** Heartworms, Macrocyclic lactones, Drug resistance, Surveillance, Genotype, Molecular markers

## Abstract

*Dirofilaria immitis* is a parasitic filarial nematode and the causative agent of heartworm disease in canids and other species. Heartworm disease is predominantly managed via macrocyclic lactone (ML) - based chemoprophylactics. Through opportunistic sampling, genotypically and phenotypically confirmed ML-resistant *D. immitis* isolates have been isolated in the Lower Mississippi River Valley region (LMRV); however, the pervasiveness of resistant isolates in the USA has not been evaluated. This study aimed to evaluate the geographic distribution and prevalence of genotypically ML-resistant heartworms in client-owned dogs across the USA over a 3-year period. Owner consent was obtained to collect microfilaremic blood samples from heartworm-positive dogs from participating clinics. Veterinarians completed a questionnaire on the known history of each dog, including treatment and travel history. A total of 310 microfilaremic blood samples were collected from 45 geographically diverse veterinary clinics located in 22 states. Microfilariae were filtered from blood, DNA extracted utilizing the QIAGEN QIAamp DNA Micro Kit and samples sequenced by the Génome Québec Innovation Centre to determine allele frequencies at nine SNP sites previously correlated with ML resistance. The highly predictive 2-SNP model was used to identify genotypically susceptible, mixed, and resistant populations. Computational analysis indicated 111 (35.8 %) were genotypically susceptible, 96 (31.0 %) were genotypically resistant, and 103 (33.2 %) were genotypically mixed. The genotypically mixed and ML-resistant infections were located within and outside of the endemic LMRV, as far north as Michigan, which indicates canine populations outside of the LMRV are at increased risk for transmission of potentially ML-resistant heartworm infections than previously hypothesized. Veterinary practitioners across the USA need to be aware of the potential risks of ML resistance heartworm infections and ensure patient compliance with recommended prevention protocols.

## Highlights

• Clinical heartworm samples collected across United States between 2021 and 2023.
• Surveillance of prevalence of SNPs correlated to ML resistance in client owned dogs.
• Evaluation of geographic distribution of SNPs correlated to ML resistance.
• One third of all collected samples 2-SNP profile correlated with ML resistance.

## Graphical abstract

**Figure:**
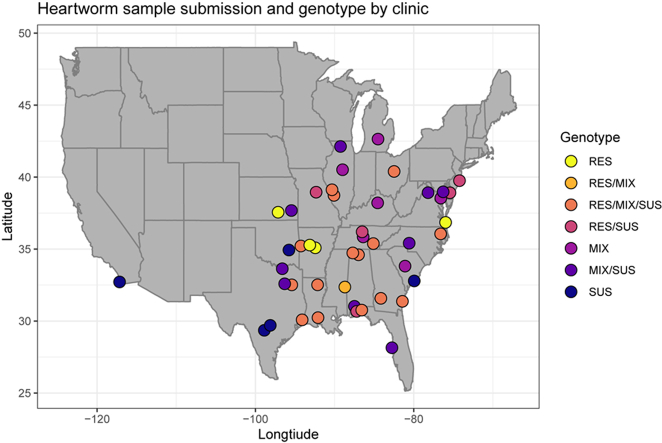

## 1 Introduction

*Dirofilaria immitis* is a parasitic filarial nematode and the causative agent of heartworm disease, a potentially fatal pulmonary infection of canids and felids. Patent infections are characterized by the colonization of the pulmonary arteries and right ventricle of the heart by adult worms. The clinical presentation is chronic and varied based on the burden of infection, with pre-patent infections often being asymptomatic. Clinical signs of a patent infection can include fatigue, shortness of breath, chronic cough, dyspnea, and cachexia. Dogs that develop caval syndrome, worms displaced into the right ventricle, atrium and/or vena cava, will acutely present with severe clinical signs (McCall et al., 2008).

Macrocyclic lactones (ML) which target the infective third-stage larvae (L3) and fourth-stage larvae (L4) are the current gold-standard chemoprophylaxis. The first ML-based medication, containing ivermectin, was approved in 1987 in the USA, followed by abamectin, milbemycin oxime, selamectin, and moxidectin (Ohishi et al., 1987a, 1987b; Wolstenholme et al., 2015; Prichard, 2021).

There have been reports of lack of efficacy (LOE) for the ML drug class against heartworm disease since 1998, mainly occurring in endemic regions of the Southeastern USA (Hampshire, 2005; Wolstenholme et al., 2015; Prichard, 2021). The number of LOE reports have continued to rise over the last 2 decades; however, it has remained challenging to determine whether the increase in LOE reports is due to potentially ML-resistant populations of *D. immitis* or to other underlying factors such as a lack of drug compliance or underdosed drug treatment (Bowman, 2012; Ballesteros et al., 2018). In 2011, ML LOE was demonstated both *in vivo* and *in vitro*, confirming the presence of potentially resistant populations circulating in North America (Blagburn et al., 2011; Bourguinat et al., 2011a, 2011b, 2015). In 2014, the heritability of true ML resistance was validated by experimentally infecting canines in laboratory settings (Pulaski et al., 2014). Overall, ML-resistant *D. immitis* isolates have been previoulsy documented from infected dogs primarily in the Lower Mississippi River Valley region (LMRV).

Whole genome analysis of *D. immitis* samples collected from the USA, Grenada, Spain, and Italy elucidated 186 potential single nucleotide polymorphism (SNP) molecular markers associated with the ML-resistant phenotype (Bourguinat et al., 2015). The top 10 SNP molecular markers which best differentiated the ML-susceptible and ML-resistant phenotypes were validated in clinical samples collected within the contiguous USA and resulted in a highly predictive 2-SNP model, SNP1: Scaffold nDi.2.2scaf00046 Positions 76278, and SNP2: Scaffold nDi.2.2scaf00046; 22857 (Bourguinat et al., 2017, 2017; Ballesteros et al., 2018). These SNP markers have since been used to assess clinical samples in North America, Europe, and Australia (Lau et al., 2021; Curry et al., 2022; Power and Šlapeta, 2022; Kumar et al., 2024; Fisher et al., 2024). SNP1 and SNP2 were further validated through the development of a highly sensitive droplet digital PCR (ddPCR) diagnostic assay (Kumar et al., 2023).

The clinical validation of these SNP molecular markers provided the first genetic tests to differentiate between ML-resistant infections and cases of opportunistic infections or owner non-compliance. However, the transmission of these ML drug resistance SNPs in the USA is not known outside the LMRV. The aim of the current study was to elucidate the broader occurrence of genotypically ML-susceptible, ML-resistant, and mixed *D. immitis* infections from client-owned dogs across the continental USA collected between August 2021 and December 2023, using the SNP molecular markers.

## 2 Material and methods

### 2.1 Clinic enrollment and patient recruitment

In total, 45 veterinary clinics were enrolled. Each study site was asked to complete an online survey which outlined general information pertaining to heartworm infections diagnosed at the clinic and detailed contact information. Following the completion of the online survey, blood collection kits were shipped to the participating veterinary clinics, and blood samples from animals which met the inclusion criteria were collected.

The inclusion criteria for enrolled cases were as follows: only client-owned dogs which were routinely care for at participating clinic could be enrolled. All dogs required a positive heartworm antigen test and the presence of circulating microfilaria (mf). All animals needed to be in good general health, with the exception of clinical signs of heartworm disease. Animals were not excluded based on breed, sex, altered status (intact, spayed, or neutered), or body weight. A minimum of 5 mL of anticoagulated whole blood in EDTA tubes was collected with the provided blood collection kits. The blood collection kits were comprised of the owner consent form, the sample documentation form, prelabelled 5 mL EDTA blood tubes, bio-labelled shipping bag, and prepaid FedEx shipping labels. The sample, along with completed owner consent and sample documentation forms, were shipped for processing within 24 h of collection. The study protocol was approved by the Zoetis Ethical Review Board, and the study was conducted in accordance with state and national/international regulations regarding animal welfare.

### 2.2 Sample processing and genomic DNA extraction

The canine venous blood samples were shipped overnight for immediate processing. A wet mount was performed to confirm the presence and count of mf in the blood (mf/mL). The mf were isolated via filtration as described by Bourguinat et al., in 2015. The venous blood was diluted 1 : 1 with a NaHCO_3_ solution, and the mf were filtered with a polycarbonate membrane filter (5.0 μm; 90 mm; Millipore Sigma, Burlington, MA, USA).

Genomic DNA (gDNA) was extracted using the QIAGEN QIAamp DNA Micro Kit (Qiagen, Valencia, CA, USA) per manufacturer instructions. The gDNA quality and quantity of each sample was assessed via NanoDrop™ One^C^ spectrophotometer (Thermo Fisher Scientific, Waltham, MA, USA) and Qubit™ (Thermo Fisher Scientific, Waltham, MA, USA). Processed gDNA samples were stored at – 20 °C prior to being plated and shipped to McGill University for sequencing.

### 2.3 SNP markers

The SNP markers used to assess the prevalence of ML resistance in client-owned dogs across the USA were the top nine SNP molecular markers clinically validated in 2018 to differentiate phenotypically ML-susceptible and ML-resistant *D. immitis* infections (Ballesteros et al., 2018).

### 2.4 Genomic DNA sequencing

Six plates were sent to the Génome Québec Innovation Centre for sequencing, containing a total of 310 samples: Plate 1 with 48, Plate 2 with 43, Plate 3 with 96, Plate 4 with 48, Plate 5 with 48, and Plate 6 with 27. The target enrichment was performed using the Fluidigm Access Array system via PCR amplification of the genomic target regions. The samples on each plate underwent parallel amplification using primers with added CS1 and CS2 tails, as described in Ballesteros et al. (2018) (Supplementary Table 1). The samples were barcoded during target enrichment which allowed for multiplexed sequencing, and adapter sequences were added during the PCR amplification reaction. Plates 1–4 were sequenced on an Illumina MiSeq Platform (Illumina Inc., San Diego, CA, USA), and Plate 5 and Plate 6 were sequenced on an Illumina NextSeq Platform (Illumina Inc., San Diego, CA, USA).

### 2.5 Data processing and analyses

The sequenced reads were aligned to the *D. immitis* reference genome nDi.2.2 (http://www.nematodes.org/genomes/dirofilaria_immitis) using BWA-mem (version 0.7.17-r1188; https://github.com/lh3/bwa) (Godel et al., 2012; Li, 2013; Howe et al., 2016, 2017). The reads were then sorted and indexed with samtools (version 1.19.2; https://github.com/samtools/samtools) and combined into a single vcf (Supplementary File 1) via bcftools (version 1.9; mpileup/merge; https://github.com/samtools/bcftools) (Li and Durbin, 2009; Danecek et al., 2021).

The allele frequencies for the nine SNP molecular markers were extracted from the vcf, requiring a threshold of at least 10 reads for inclusion in downstream analyses. Every SNP containing loci in each clinical sample were categorized based on the frequency of the alternate allele as follows: susceptible (≤16.9); mixed (17.0–22.9); or resistant (≥23. 0) (Ballesteros et al., 2018; Curry et al., 2022; Fisher et al., 2024). The genetic profile of each sample was evaluated using the highly predictive and clinically validated 2-SNP model (Ballesteros et al., 2018). Data was processed using R (https://www.R-project.org), reading in the combined vcf with vcfR (https://github.com/knausb/vcfR) and visuals were created via ggplot2 (version 3.4.2; https://github.com/tidyverse/ggplot2) and/or ggbeeswarm (version 0.7.2; https://github.com/eclarke/ggbeeswarm) (Wickham et al., 2016; Knaus and Grünwald, 2017; R Core Team, 2021; Clarke et al., 2023). An R script detailing the calculation of SNP frequencies, classification and plotting is attached (Supplementary File 2).

## 3 Results

### 3.1 Distribution of samples

Forty-five clinics were enrolled in the current study. Clinics were geographically spread across 22 states, with most clinics located in the LMRV. Heartworm positive blood samples were received from participating clinics in 19 states in 2021, 13 states in 2022, and 9 states in 2023. The majority of samples were received from Alabama, Georgia, and Texas, which made up 51.6 % of all samples collected. In total 310 samples were collected over the three-year collection period, 110 samples between August and December 2021, 128 samples in 2022, and 72 samples in 2023 (Table 1).

**Table 1:** The sample demographics of the canine hosts during the three-year collection period between 2021 and 2023.

Collection Year	Sex		Age^a^					Altered Status^b^				Historical^c^	
	♀	♂	0–3	4–6	7–9	10–12	≥13	♀ Intact	♀ Spayed	♂ Intact	♂ Neutered	Travel	Preventative
**2021**	64	46	51	32	14	10	2	25	34	24	30	11	20
**2022**	54	74	47	48	16	14	3	28	25	34	40	9	12
**2023**	29	43	31	20	12	6	1	8	19	27	13	3	3

a Age was provided for 307 of the 310 samples collected in the sample documentation form.

b Altered status was provided for 307 of the 310 samples collected in the sample documentation form.

c Historical data based on owner disclosure in the sample documentation form.

### 3.2 Sample/patient demographics

A sample documentation form was provided in the blood collection kits shipped to participating veterinary clinics. Detailed patient demographics were collected including breed, sex, altered status (intact, spayed, or neutered), age, travel history, treatment history, etc. (Table 1, Supplementary File 3).

There was a slightly uneven sex split with 47.4 % of host animals being female and 52.6 % being male (Table 1). The infected animals skewed younger with 73.9 % of dogs being under the age of six years (Table 1). Of the 310 samples collected only 35 animals were reported to be on a heartworm preventative at the time of diagnosis, this included: Advantage Multi®, Heartgard Plus®, Interceptor®, Interceptor® Plus, Iverhart Max®, Iverhart Plus®, Nexgard Spectra®, ProHeart® 12, Sentinel® Spectrum®, Simparica Trio™, Trifexis®, and Tri-Heart® Plus.

### 3.3 SNP molecular markers

**Fig. 1:**
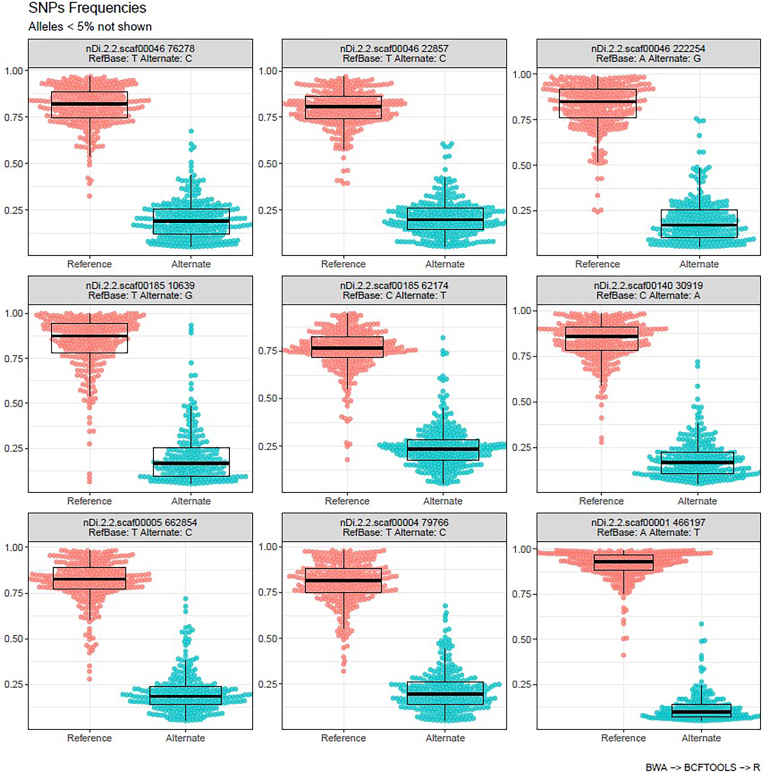
Alternate allele frequency of the 310 USA clinical samples at the nine SNP molecular markers of interest. The red circles represent the reference base for each SNP molecular marker, and the blue circles represent the alternate base for each SNP molecular marker. The alternative allele frequencies were prepared in comparison to the *D. immitis* reference genome nDi.2.2.

All 310 samples were processed for gDNA extraction and assessed for quantity and quality (Supplementary File 3). These samples were sent to the Génome Québec Innovation Centre for amplicon sequencing surrounding the nine SNP markers of interest. The reads from each sample were aligned to the *D. immitis* reference genome. The base frequencies for the nine SNP positions were extracted and assimilated to the allele frequencies (Fig. 1).

**Fig. 2:**
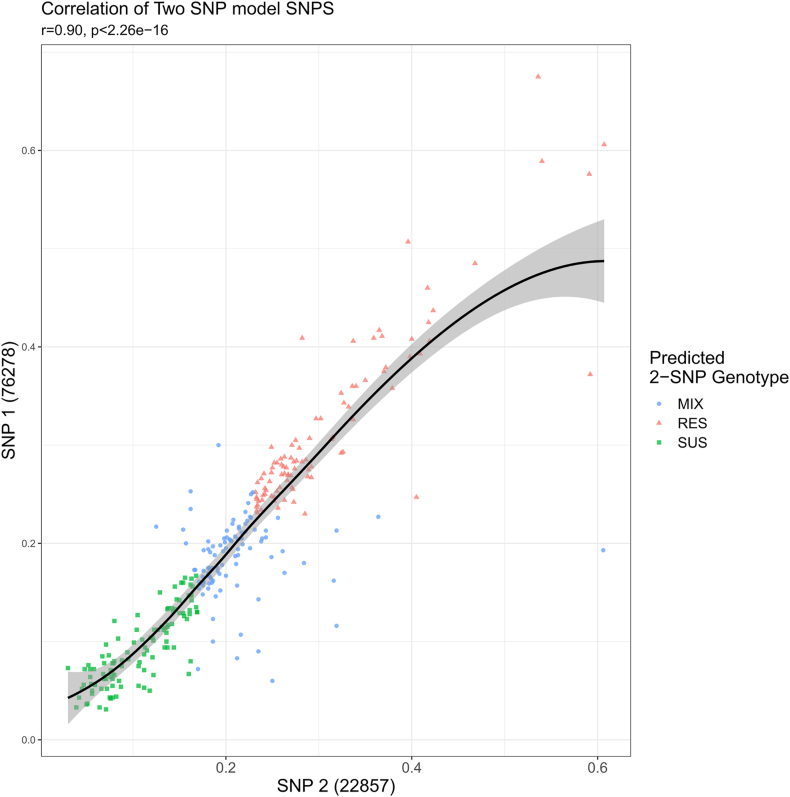
Predictive correlation of the top 2 SNP markers (SNP1: Scaffold nDi.2.2scaf00046 Positions 76278, and SNP2: Scaffold nDi.2.2scaf00046; 22857) of the 310 USA clinical samples. The green squares represent genotypically ML-susceptible samples (SUS), the red triangles represent genotypically ML-resistant samples (RES), and the blue circles represent genotypically mixed samples (MIX).

The predicted ML-susceptibility genotype of each sample was called using the clinically validated 2-SNP model: SNP1: Scaffold nDi.2.2scaf00046 Positions 76278, and SNP2: Scaffold nDi.2.2scaf00046; 22857 (Ballesteros et al., 2018). If the SNP markers were called SUS: SUS the sample was called as ML-susceptible, if the SNP markers were called as RES: RES the sample was called as ML-resistant, all other combinations were called as genotypically mixed (Supplementary File 3). The frequencies of the alternate allele at these positions were highly correlative to the genotypic ML susceptibility profile with an R-value of 0.90 (Fig. 2).

**Fig. 3:**
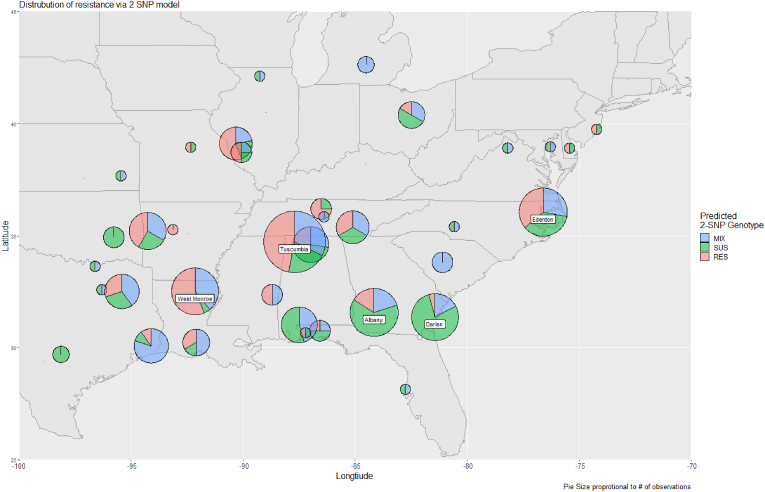
Distribution of the 310 USA clinical samples collected between 2021 and 2023, with pie sizes proportional to the number of observations per clinic. Clinics with less than three observations did not meet the inclusion cut-off. Clinics with greater than 20 observations are further identified by town name. The predicted genotypic distribution presented is spatially contiguous and encompasses ≥99 % of the data, with clinics from California contributing ≤1 %. The green represents genotypically ML-susceptible samples (SUS), the red represents genotypically ML-resistant samples (RES), and the blue represents genotypically mixed samples (MIX).

In total 111 samples were genotypically ML-susceptible, 96 were genotypically ML-resistant, and 103 were genotypically mixed based on the predictive 2-SNP model (Table 2). Fig. 3 demonstrates the distribution of the veterinary practices across the contiguous United States and the types of samples received from each clinic based on their predictive genotypic profile. The geographic distribution of the ML-resistant and mixed samples was not limited to the LMRV and were seen as far north as Michigan and as far east as New Jersey.

**Table 2:** The predicted macrocyclic lactone susceptibility profile based on the 2-SNP model for samples collected from 22 states during the three-year collection period between 2021 and 2023.

State	2-SNP Genotype			Samples per State	Percent of Total Sample (%)
	RES	MIX	SUS		
**AL**	32	28	24	84	27.1
**AR**	8	4	3	15	4.8
**CA**	0	0	2	2	0.6
**DE**	1	0	1	2	0.6
**FL**	2	2	4	8	2.6
**GA**	5	9	34	48	15.5
**IL**	7	5	4	16	5.2
**KS**	1	1	1	3	1.0
**KY**	0	1	0	1	0.3
**LA**	15	12	2	29	9.4
**MD**	0	2	1	3	1.0
**MI**	0	3	0	3	1.0
**MO**	1	0	1	2	0.6
**MS**	2	2	0	4	1.3
**NC**	9	8	10	27	8.7
**NJ**	1	0	1	2	0.6
**OH**	1	2	3	6	1.9
**OK**	0	0	4	4	1.3
**SC**	0	4	1	5	1.6
**TN**	6	5	4	15	4.8
**TX**	4	14	10	28	9.0
**VA**	1	1	1	3	1.0
**Total**	96	103	111	310	100.0

## 4 Discussion

The geographic distribution and number of heartworm infections in North America continues to rise due to climate change, reservoir populations, and human factors, such as the transportation of pets and rescue animals across state and international borders (Drake and Wiseman, 2018; Jacobson et al., 2020; AHS, 2022). *D*. *immitis* isolates resistant to ML-based chemoprophylaxis have been confirmed to be circulating in endemic regions of the Southeastern USA, such as the LMRV (Pulaski et al., 2014; Bourguinat et al., 2015; Ballesteros et al., 2018; Fisher et al., 2024). These ML-resistant populations have been confirmed via a combination of phenotypic and genotypic testing, which includes mf suppression testing and MiSeq sequencing of the clinically validated and highly predictive 2-SNP model. The present research represents the first multi-state and multi-year investigation into the broader occurrence of genotypically ML-resistant *D. immitis* infections using the 2-SNP model.

Between August 2021 and December 2023, a total of 310 *D. immitis* positive blood samples were collected from 45 clinics across 22 USA states. In 13 of the 22 states surveyed, heartworm infections predicted to be ML-resistant based on the 2-SNP model were identified. Moreover, the genotypically mixed and ML-resistant samples made up 33.2 % and 31.0 % of the total samples, respectively (Fig. 3). Overall, genotypically mixed and ML-resistant infections were located within and outside of the endemic LMRV, including states outside of the high endemic Southeast region.

Interestingly, previous surveillance of 96 samples from client-owned dogs in 11 counties in Southeast Missouri were assessed as 94.8 % ML-resistant and 4.2 % genotypically mixed based on F_ST_ calculations (Fisher et al., 2024). In comparison, the samples collected from Missouri in the current study were assessed as 50 % ML-resistant and 50 % ML-susceptible; however, the sample collection was limited to two client-owned dogs in Boone County in the Central Hills region. The observed difference in the proportion of genotypically ML-resistant infections between the current study and Fisher et al. (2024) may be the result of more northern sampling and/or the lower statistical power when comparing 96 samples to two samples. Moreover, Southeastern Missouri may have a higher incidence of heartworm disease due to its humid wetland terrain, which is highly conducive to mosquito breeding when compared to the Central Hills region.

These results demonstrate that SNPs associated with ML resistance are widespread in the US. In contrast, genotypic assessment of European clinical samples collected from Greece, Romania, Italy, Hungary, and Spain using the 2-SNP predictive model did not provide genomic evidence of ML resistance (Curry et al., 2022; Kumar et al., 2024). It is important to consider that the global *D. immitis* populations have been shown to be quite genetically diverse. Moreover, the recent transportation of a heartworm infected dog from the USA to Italy with circulating ML-resistant mf, identified using the 2-SNP model, highlights the potential risk for ML resistance to spread outside of North America as a result of the transportation of animals across international borders (Traversa et al., 2024).

It should be noted that a disproportionate number of these samples came from highly endemic regions such as Alabama, Georgia, Louisiana, North Carolina, and Texas. In particular, samples originating from Alabama, Georgia, and Texas accounted for more than 50 % of the total samples received (Table 2). More sampling in emerging hot spots such as the Southwest and the Upper Midwest would provide additional clarity on the proportionality of genotypically mixed and ML-resistant populations circulating within these regions over time.

The clinical samples collected over the three-year collection period were all single point blood draws. As a result, unlike Ballesteros et al. (2018), there was no matched phenotypic mf suppression test data to correlate with the genotypic sequencing results. This is an important consideration as genotypic ML susceptibility may not correlate one to one with phenotypic ML susceptibility. *D. immitis* populations, unlike clonal bacteria colonies, are heterogeneous and can have a degree of genetic variability within an individual host. Currently, *D. immitis* isolates are characterized as ML-susceptible due to their proposed lack of ML exposure and/or the prevention of a patent infection when treated with ML chemoprophylaxis at the proposed commercial dose rate and mf suppression testing. As a result, ML resistance is currently defined as less than 100 % efficacy in *in vivo* studies. Notably, as heartworm infections continue to spread across the USA, it will become increasingly difficult to find truly naïve ML-susceptible *D. immitis* isolates. A lack of history of ML treatment in a particular host does not mean the ancestors of the challenge population were not exposed to repeated ML chemoprophylaxis. *D. immitis* populations may appear as ML-susceptible or ML-resistant at a given dose rate but may show a shift in their phenotypic ML susceptibility profile should a dose-response curve be investigated, multiple doses of ML be administered, or a more potent ML preventatives, such as moxidectin, be used (Bowman et al., 2017; McTier et al., 2017, 2021; Kryda et al., 2019; Prichard and Geary, 2019; Myers et al., 2022; Savadelis et al., 2022).

The genotypes of the *D. immitis* samples collected in the current longitudinal study were characterized based on the 2-SNP model composed of SNP1: Scaffold nDi.2.2scaf00046 Positions 76278, and SNP2: Scaffold nDi.2.2scaf00046; 22857 (Ballesteros et al., 2018). These SNP markers were first identified on the nDi.2.2 reference genome and have since been localized at positions SNP 1: 8326593 and SNP 2: 8273382 on chromosome 3 of the WSI_2.2 chromosome level assembly (Gandasegui et al., 2024). Further analysis of this region on chromosome 3, along with matched phenotypic data, may help to elucidate functional changes associated with ML resistance. A whole genome sequencing approach in future surveillance studies would allow for the analysis of the 2-SNP model, changes to chromosome 3, and the identification of any novel markers of ML resistance that may have arisen in the field over the last decade, particularly if ML resistance is multigenic.

## 5 Conclusion

It had previously been thought that ML resistance in *D. immitis*, and the associated risks of infection with these populations, was primarily confined to the LMRV. This study demonstrates the presence of genotypically mixed and ML-resistant infections within and outside of the LMRV, including areas outside of the high endemic Southeast region of the country, as far north as Michigan and as far east as New Jersey. The three MIX samples from Michigan were identified in animals originating from Georgia and Arkansas, and the RES New Jersey sample originated from Virginia. These finding further highlight that the translocation of dogs from high endemic regions can lead to the introduction and potentially the spread of genotypically mixed and ML-resistant isolates. The transmission of heartworm disease has continued despite the availability of preventives over nearly four decades, and veterinary practitioners across the USA should take the potential risks of ML resistance into consideration when diagnosing heartworm infections in apparently compliant patients and when making heartworm preventive recommendations.

## CRediT authorship contribution statement

**Emily Curry:** Writing – review & editing, Writing – original draft, Validation, Formal analysis, Data curation. **David Tack:** Writing – review & editing, Visualization, Software, Formal analysis. **Jessica Rodriguez:** Writing – review & editing, Methodology, Conceptualization. **Danielle Brehm-Lowe:** Writing – review & editing, Investigation, Data curation. **John Letherer:** Investigation. **Megan Lineberry:** Writing – review & editing, Data curation. **Roger Prichard:** Writing – review & editing, Methodology, Investigation, Data curation, Conceptualization. **Tobias Clark:** Writing – review & editing, Validation, Supervision, Project administration, Methodology, Funding acquisition, Conceptualization.

## Ethics approval

The study protocol was reviewed and approved by the Zoetis Ethical Review Board, and the study was conducted in accordance with state and national/international regulations regarding animal welfare.

## Data statement

The data presented in this article is openly available on the NCBI Sequence Read Archive as fastq files under BioProject PRJNA1289889. Vcf files and additional data is available in the supplementary files.

## Declaration of competing interest

The authors declare the following financial interests/personal relationships which may be considered as potential competing interests:Emily Curry, David Tack, Jessica Rodriguez, Danielle Brehm-Lowe, John Letherer, Megan Lineberry and Tobias Clark reports a relationship with Zoetis Inc. that includes: employment. Roger Prichard received financial support for the genotype analysis from Zoetis Inc.

## Abbreviations

• ddPCR: Droplet digital PCR
• gDNA: Genomic DNA
• LMRV: Lower Mississippi River Valley
• LOE: Lack of efficacy
• ML: Macrocyclic lactones
• mf: Microfilaria
• SNP: Single nucleotide polymorphism

## References

[bib1] AHS (2022). Heartworm incidence maps. https://www.heartwormsociety.org/veterinary-resources/incidence-maps.

[bib2] Ballesteros C., Pulaski C.N., Bourguinat C., Keller K., Prichard R.K., Geary T.G. (2018). Clinical validation of molecular markers of macrocyclic lactone resistance in *Dirofilaria immitis*. Int. J. Parasitol. Drugs Drug Resist.

[bib3] Blagburn B.L., Dillon A.R., Arther R.G., Butler J.M., Newton J.C. (2011). Comparative efficacy of four commercially available heartworm preventive products against the MP3 laboratory strain of *Dirofilaria immitis*. Vet. Parasitol..

[bib4] Bourguinat C., Keller K., Bhan A., Peregrine A., Geary T., Prichard R. (2011). Macrocyclic lactone resistance in *Dirofilaria immitis*. Vet. Parasitol..

[bib5] Bourguinat C., Keller K., Prichard R.K., Geary T.G. (2011). Genetic polymorphism in *Dirofilaria immitis*. Vet. Parasitol..

[bib6] Bourguinat C., Lee A.C.Y., Lizundia R., Blagburn B.L., Liotta J.L., Kraus M.S., Keller K., Epe C., Letourneau L., Kleinman C.L., Paterson T., Gomez E.C., Montoya-Alonso J.A., Smith H., Bhan A., Peregrine A.S., Carmichael J., Drake J., Schenker R., Kaminsky R., Bowman D.D., Geary T.G., Prichard R.K. (2015). Macrocyclic lactone resistance in *dirofilaria Immitis*: failure of heartworm preventives and investigation of genetic markers for resistance. Vet. Parasitol..

[bib7] Bourguinat C., Keller K., Xia J., Lepage P., McTier T.L., Woods D.J., Prichard R.K. (2017). Genetic profiles of ten *Dirofilaria immitis* isolates susceptible or resistant to macrocyclic lactone heartworm preventives. Parasites Vectors.

[bib8] Bourguinat C., Lefebvre F., Sandoval J., Bondesen B., Moreno Y., Prichard R.K. (2017). *Dirofilaria immitis* JYD-34 isolate: whole genome analysis. Parasites Vectors.

[bib9] Bowman D.D. (2012). Heartworms, macrocyclic lactones, and the specter of resistance to prevention in the United States. Parasites Vectors.

[bib10] Bowman D.D., McTier T.L., Adams E.L., Mahabir S.P., Login J.A., Bidgood T., Woods D.J. (2017). Evaluation of the efficacy of ProHeart 6 (Moxidectin) against a resistant isolate of *Dirofilaria immitis* (JYD-34) in dogs. Parasites Vectors.

[bib11] Clarke E., Sherrill-Mix S., Dawson C., Clarke M.E. (2023).

[bib12] Curry E., Traversa D., Carretón E., Kramer L., Sager H., Young L., Prichard R. (2022). *Dirofilaria immitis*: genotyping randomly selected European clinical samples and USA laboratory isolates with molecular markers associated with macrocyclic lactone susceptibility and resistance. Pathogens.

[bib13] Danecek P., Bonfield J.K., Liddle J., Marshall J., Ohan V., Pollard M.O., Whitwham A., Keane T., McCarthy S.A., Davies R.M., Li H. (2021). Twelve years of SAMtools and BCFtools. GigaScience.

[bib14] Drake J., Wiseman S. (2018). Increasing incidence of *Dirofilaria immitis* in dogs in USA with focus on the southeast region 2013–2016. Parasites Vectors.

[bib15] Fisher P.T., Keller K., Prichard R.K. (2024). Investigating *Dirofilaria immitis* isolates infecting domestic canines and their susceptibility/resistance patterns to macrocyclic lactones in the northern region of the Mississippi Delta area (southeast Missouri). Vet. Parasitol..

[bib16] Gandasegui J., Power R.I., Curry E., Lau D.C.W., O'Neill C.M., Wolstenholme A., Prichard R., Šlapeta J., Doyle S.R. (2024). Genome structure and population genomics of the canine heartworm *Dirofilaria immitis*. Int. J. Parasitol..

[bib17] Godel C., Kumar S., Koutsovoulos G., Ludin P., Nilsson D., Comandatore F., Wrobel N., Thompson M., Schmid C.D., Goto S., Bringaud F. (2012). The genome of the heartworm, *Dirofilaria immitis*, reveals drug and vaccine targets. FASEB J..

[bib18] Hampshire V.A. (2005). Evaluation of efficacy of heartworm preventive products at the FDA. Vet. Parasitol..

[bib19] Howe K.L., Bolt B.J., Cain S., Chan J., Chen W.J., Davis P., Done J., Down T., Gao S., Grove C., Harris T.W. (2016). WormBase 2016: expanding to enable helminth genomic research. Nucleic Acid..

[bib20] Howe K.L., Bolt B.J., Shafie M., Kersey P., Berrimen M. (2017). WormBase ParaSite – a comprehensive resource for helminth genomics. WormBase ParaSite – a comprehensive resource for helminth genomics. Mol. Biochem. Parasitol..

[bib21] Jacobson L.S., Ward K.A., Lacaden A.B., Hornak T.A. (2020). Prevalence of heartworm in relocated, local and outreach clinic dogs: a Canadian sheltering perspective. Vet. Parasitol..

[bib22] Knaus B.J., Grünwald N.J. (2017). vcfr: a package to manipulate and visualize variant call format data in R. Mol. Ecol. Resour.

[bib23] Kumar S., Prichard R.K., Long T. (2023). Droplet digital PCR as a tool to detect resistant isolates of *Dirofilaria immitis*. Int. J. Parasitol. Drugs Drug Resist.

[bib24] Kumar S., Che H., Chiummo R., Heuer L., Schneider C., Werr M., Guerino F., Papadopolous E., Diakou A., Mihalca A.D., Traversa D. (2024). Genotyping USA laboratory-maintained isolates and European clinical isolates of *Dirofilaria immitis* to assess macrocyclic lactone susceptibility or resistance at predictive SNP sites using droplet digital PCR. Vet. Parasitol..

[bib25] Kryda K., Six R.H., Walsh K.F., Holzmer S.J., Chapin S., Mahabir S.P. (2019). Laboratory and field studies to investigate the efficacy of a novel, orally administered combination product containing moxidectin, sarolaner and pyrantel for the prevention of heartworm disease (Dirofilaria immitis) in dogs. Parasites Vectors12.

[bib26] Lau D.C.-W., McLeod S., Collaery S., Peou S., Tran A.T., Liang M., Šlapeta J. (2021). Whole-genome reference of *Dirofilaria immitis* from Australia to determine single nucleotide polymorphisms associated with macrocyclic lactone resistance in the USA. CRPVBD.

[bib27] Li H., Durbin R. (2009). Fast and accurate short read alignment with burrows–wheeler transform. Bioinformatics.

[bib28] Li H. (2013).

[bib29] McCall J.W., Genchi C., Kramer L.H., Guerrero J., Venco L. (2008). Advances in Parasitology.

[bib30] McTier T.L., Six R.H., Pullins A., Chapin S., McCall J.W., Rugg D., Maeder S.J., Woods D.J. (2017). Efficacy of oral moxidectin against susceptible and resistant isolates of *Dirofilaria immitis* in dogs. Parasites Vectors.

[bib31] McTier T.L., Holzmer S., Kryda K., Mahabir S., McCall J.W., Trombley J., Maeder S.J. (2021). Comparative preventive efficacy of ProHeart 12, heartgard plus and interceptor plus against a macrocyclic lactone-resistant strain (JYD-34) of heartworm (*Dirofilaria immitis*) in dogs. Parasites Vectors.

[bib32] Myers J.A., Holzmer S., McCall J.W., Mahabir S.P., McTier T.L., Maeder S.J., Kryda K. (2022). Preventive efficacy of six monthly oral doses of simparica trio, heartgard plus, and interceptor plus against a macrocyclic lactone-resistant strain (ZoeLA) of heartworm (*Dirofilaria immitis*) in dogs. Parasites Vectors.

[bib33] Ohishi I., Katae H., Hayasaki M., Nakagaki K., Tada Y. (1987). Prophylactic activity of ivermectin against *Dirofilaria immitis* infection in dogs: establishment of effective dose and administration schedule. Jpn. J. Vet..

[bib34] Ohishi I., Katae H., Hayasaki M., Tada Y. (1987). Prophylactic activity of ivermectin against *Dirofilaria immitis* infection in dogs: larvicidal activity of ivermectin against *D. immitis* larvae 30 days after infection. Jpn. J. Vet..

[bib35] Power R.I., Šlapeta J. (2022). Exploration of the sensitivity to macrocyclic lactones in the canine heartworm (*Dirofilaria immitis*) in Australia using phenotypic and genotypic approaches. Int. J. Parasitol.: Drugs Drug Resist..

[bib36] Prichard R.K., Geary T.G. (2019). Perspectives on the utility of moxidectin for the control of parasitic nematodes in the face of developing anthelmintic resistance. Int. J. Parasitol.: Drugs Drug Resist..

[bib37] Prichard R.K. (2021). Macrocyclic lactone resistance in *dirofilaria Immitis*: risks for prevention of heartworm disease. Invited review Int. J. Parasitol..

[bib38] Pulaski C.N., Malone J.B., Bourguinat C., Prichard R., Geary T., Ward D., Klei T.R., Guidry T., Smith G.B., Delcambre B., Bova J. (2014). Establishment of macrocyclic lactone resistant *Dirofilaria immitis* isolates in experimentally infected laboratory dogs. Parasites Vectors.

[bib39] R Core Team (2021).

[bib40] Savadelis M.D., McTier T.L., Kryda K., Maeder S.J., Woods D.J. (2022). Moxidectin: heartworm disease prevention in dogs in the face of emerging macrocyclic lactone resistance. Parasites Vectors.

[bib41] Traversa D., Diakou A., Colombo M., Kumar S., Long T., Chaintoutis S.C., Venco L., Miller G.B., Prichard R. (2024). First case of macrocyclic lactone-resistant *Dirofilaria immitis* in europe-cause for concern. Int. J. Parasitol.: Drugs Drug Resist..

[bib42] Wickham H., Chang W., Wickham M.H. (2016). Package ‘ggplot2’. Create elegant data visualisations using the grammar of graphics. Version.

[bib43] Wolstenholme A.J., Evans C.C., Jimenez P.D., Moorhead A.R. (2015). The emergence of macrocyclic lactone resistance in the canine heartworm, *Dirofilaria immitis*. Parasitology.

