# Supplementary material for: Surveillance of single nucleotide polymorphisms correlated to macrocyclic lactone resistance in Dirofilaria immitis from client-owned dogs across the United States
Source: Int J Parasitol Drugs Drug Resist. 2025 Aug 5;29:100604. doi: 10.1016/j.ijpddr.2025.100604 (PMC12355919; doi:10.1016/j.ijpddr.2025.100604)
Supplement: Multimedia component 4 [file mmc4.docx]

Supplementary Table 1: SNP ID position and primer sequences as previously described by Ballesteros et al., 2018.

| SNP ID | Scaffold | Position | Primer Sequence |
| --- | --- | --- | --- |
| SNP 1 | nDi.2.2.scaf00046 | 76278 | scaf00046-76278-CS1F ACACTGACGACATGGTTCTACAGGCCAATAAATAAAGGCTA scaf00046-76278-CS2R TACGGTAGCAGAGACTTGGTCTGTTTTCTGGAATTATCAGAC |
| SNP 2 | nDi.2.2.scaf00046 | 22857 | scaf00046-22857-CS1F ACACTGACGACATGGTTCTACACGAGGTAAAGCACACAGAAG scaf00046-22857-CS2R TACGGTAGCAGAGACTTGGTCTCAACAAAATGCCGCAGATGG |
| SNP 3 | nDi.2.2.scaf00046 | 222254 | scaf00046-222254-CS1F ACACTGACGACATGGTTCTACACATCGTTGTCAACTTCCTGC scaf00046-222254-CS2R TACGGTAGCAGAGACTTGGTCTGAAATTTGAAAATGGGTACT |
| SNP 4 | nDi.2.2.scaf00185 | 10639 | scaf00185-10639-CS1F ACACTGACGACATGGTTCTACAACGCAGGAAAGCTTTAATGG scaf00185-10639-CS2R TACGGTAGCAGAGACTTGGTCTATCATCATTTTATCAATTCC |
| SNP 5 | nDi.2.2.scaf00185 | 62174 | scaf00185-62174-CS1F ACACTGACGACATGGTTCTACATCGATCATTTAGTAACAACG scaf00185-62174-CS2R TACGGTAGCAGAGACTTGGTCTTTGCGTTACAGCGCCAAATC |
| SNP 6 | nDi.2.2.scaf00140 | 30919 | scaf00140-30919-CS1F ACACTGACGACATGGTTCTACACGAAGAAGAAACTTTTCGGG scaf00140-30919-CS2R TACGGTAGCAGAGACTTGGTCTGTACAATTAATTGCTGTTCGC |
| SNP 7 | nDi.2.2.scaf00005 | 662854 | scaf00005-662854-CS1F ACACTGACGACATGGTTCTACAGTTATTTGCACTACTCTCCC scaf00005-662854-CS2R TACGGTAGCAGAGACTTGGTCTTGGCGTACTGATCACATTGG |
| SNP 8 | nDi.2.2.scaf00004 | 79766 | scaf00004-79766-CS1F ACACTGACGACATGGTTCTACACGTGACTAAAAGAATAGTG scaf00004-79766-CS2R TACGGTAGCAGAGACTTGGTCTCAATTTAGGGATATGACACAG |
| SNP 9 | nDi.2.2.scaf00001 | 466197 | scaf00001-466197-CS1F ACACTGACGACATGGTTCTACATTCTATCGAAAACCTTCCAG scaf00001-466197-CS2R TACGGTAGCAGAGACTTGGTCTAGGTTGCAAAAGTTGCAATG |
